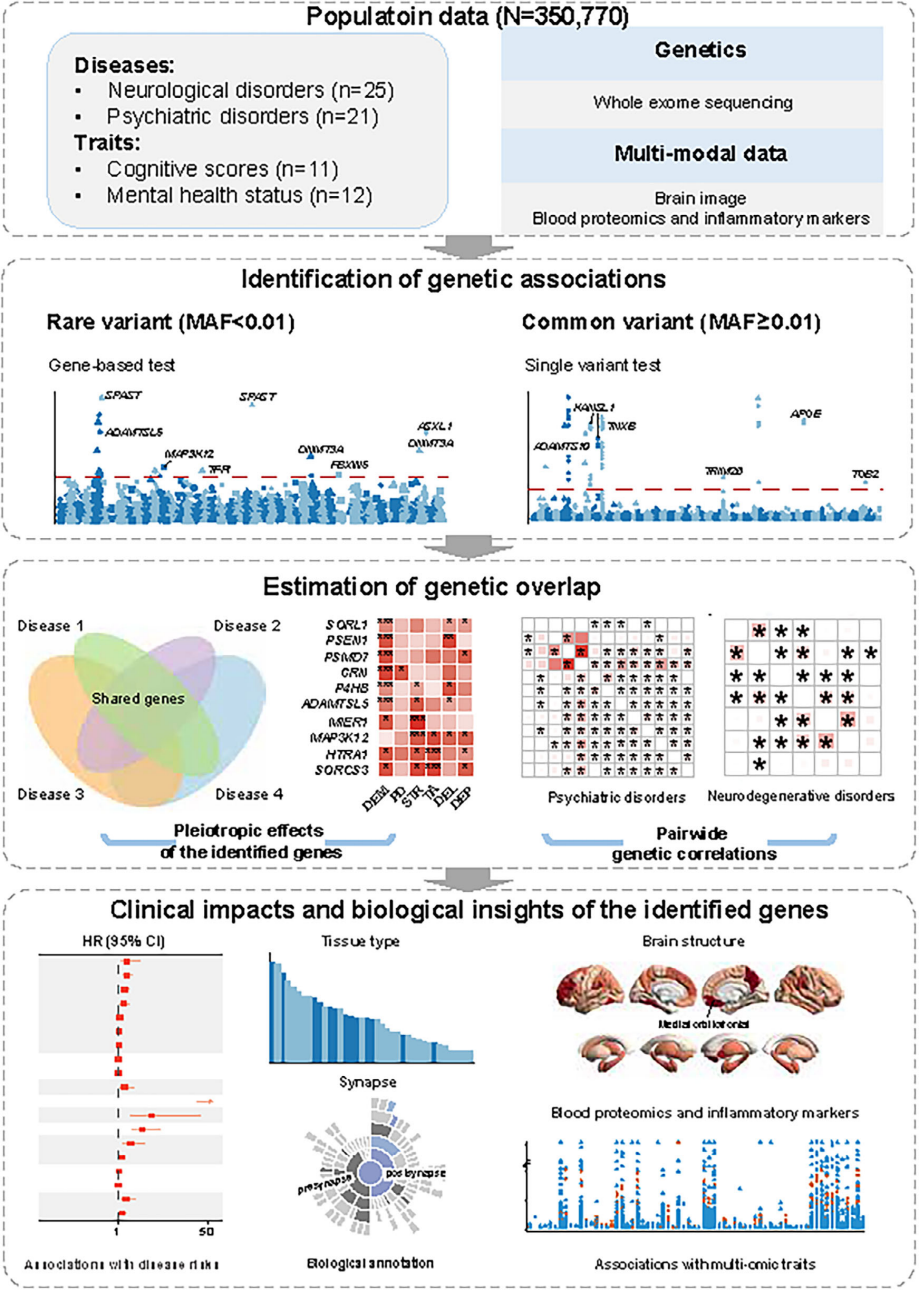# Large‐scale whole exome analysis identifies genes for dementia and genetic correlation with neuropsychiatric traits

**DOI:** 10.1002/alz70855_103063

**Published:** 2025-12-24

**Authors:** Yue‐Ting Deng, Bang‐Sheng Wu, Jin‐tai Yu

**Affiliations:** ^1^ Huashan hospital, Fudan University, Shanghai, China

## Abstract

**Background:**

While numerous genomic loci have been identified for dementia, the contribution of protein‐coding variants and the genetic correlations between dementia and neuropsychiatric traits have yet to be determined.

**Method:**

Here, we conducted a large‐scale whole‐exome‐sequencing study to interrogate the impact of protein‐coding variants on dementia and other 68 neuropsychiatric traits (containing 45 neuropsychiatric diseases and 23 cognition and mental health measures) in 350,770 adults from the UK Biobank. Pairwise estimation of genetic correlations between dementia and neuropsychiatric traits at coding‐variant level were then conducted using burden heritability regression analysis. Finally, a comprehensive multi‐omics analysis was performed to investigate whether alterations in brain structures, blood proteins and inflammation potentially contribute to the gene‐phenotype linkages.

**Result:**

Twenty novel genes were associated with neuropsychiatric diseases through coding variants, among which 14 genes had impacts on the longitudinal risks of diseases. Specially, gene‐based analysis identified seven genes for dementia, among which four (ADAMTSL5, P4HB, PSMD7, and LMTK3) were novel. Thirty novel genes were associated with cognition and mental health measures, with SYNGAP1 showing pleiotropic effects across cognitive function domains. Burden heritability regression highlighted shared genetic associations among dementia and stroke, neurodegenerative diseases and some mental disorders. Moreover, we found significant genetic correlations within neurodegenerative and psychiatric disorders at whole‐exome level, but not between neurological and psychiatric disorders. The multi‐omics analysis revealed that 29 identified genes were consistently correlated with the right medial orbitofrontal cortex, implicating its potential role in neuropsychiatric conditions.

**Conclusion:**

Our findings characterized a compendium of protein‐coding variants for future research on the biology and therapeutics of neuropsychiatric phenotypes including dementia.